# Contact, moral foundations or knowledge? What predicts attitudes towards women who undergo IVF

**DOI:** 10.1186/s12884-021-03810-9

**Published:** 2021-05-01

**Authors:** Alicja Malina, Marta Roczniewska, Julie Ann Pooley

**Affiliations:** 1 Faculty of Psychology, Kazimierz Wielki University, Bydgoszcz, Poland; 2 Faculty of Psychology in Sopot, SWPS University of Social Sciences and Humanities, Warszawa, Poland; 3 Department of Learning, Informatics, Management and Ethics, Karolinska Institute, Stockholm, Sweden; 4 School of Arts and Humanities, Edith Cowan University, Perth, Western Australia

**Keywords:** In vitro fertilization, Infertility, Attitudes, Contact hypothesis, Moral foundations

## Abstract

**Background:**

The willingness to try in vitro fertilization (IVF) as an infertility treatment, as well as its psychosocial consequences for couples, may be influenced by how they perceive the attitudes of general public towards this procedure. The focus of the current study was to identify predictors of attitudes towards mothers who underwent IVF to conceive a child. Three predictors were derived from attitude components: contact with someone who had undergone IVF (behavior), moral foundations (emotions), and the level of knowledge (cognition) about IVF.

**Method:**

In total, 817 participants (118 male and 692 female, 7 unreported) from Poland took part in the study. Participants were asked whether they knew a person who underwent IVF, completed a *Moral Foundation Questionnaire, *and answered a pre-piloted IVF knowledge test*.* Attitudes towards women who utilised IVF were measured with a modified *Bogardus Social Distance Scale*. Data were analysed using hierarchical and logistic regression analyses.

**Results:**

The results showed that there was a weak link between previous contact with a person who underwent IVF and a positive attitude toward a woman who underwent IVF. The attitudes was also predicted by moral foundations: positively by care/harm and fairness/cheating foundations, and negatively by sanctity/degradation. Importantly, more knowledge about IVF was linked with a more positive attitude towards IVF, and this effect explained additional variance over and above moral foundations.

**Conclusions:**

Our study implies the need of psychoeducation to prevent stigmatization of individuals who try IVF due to infertility.

## Background

Infertility issues and the threat of childlessness is a situation many couples struggle with globally. Research argues that infertility can cause serious harm to couple’s well-being, as becoming a parent is an important part of adult social role and identity [1]. One of the main methods of assisted reproduction used in many countries is in vitro fertilization (IVF). Despite well-documented psychological difficulties related to undertaking the IVF procedure, this method is common and popular due to the reported success rates [2]*.* However, the willingness to try IVF and its psychosocial consequences for couples may depend on how the couples perceive the attitudes of people around them, attitudes of the general public, and the social support they receive. These attitudes may influence both the course of treatment and further functioning of the couples [3]. The aim of this study was, therefore, to better understand the sources of attitudes towards people who undergo IVF. For this purpose, we investigated the roles of previous contact, moral codes, and knowledge about IVF in forming attitudes towards people who seek IVF.

Over the past three decades rates of positive attitude towards couples using the IVF procedure have been systematically increasing. Even though IVF has been available in Poland for the past 30 years, there are clashing public opinions about the legitimacy and acceptance of using IVF as a method to treat infertility [4, 5] While the acceptance for using IVF by infertile married couples has increased from 60% in 2008 to 79% in 2012 [6], this result is still far below the acceptance rates of other countries in Western Europe [7].

Different aspects of social negative assessment of the IVF procedure may intensify the feeling of loss, shame and social mismatch that often accompanies infertility [8–10]*.* Medical and psychological staff involved in the IVF process indicate psychological aspects of infertility treatment has not yet been well recognized [11]. As argued by Bronfenbrenner [12], families are nested in many environments and it is recognised that the influence of public attitude and policy on the functioning of the family and the members within it. Given that the age of the oldest person conceived by IVF is only 42 years old, there is still limited knowledge globally about the differing attitudes, effects for and of families that have chosen and navigated the IVF process. This would present an opportunity to explore IVF attitudes in the general public and the potential relationship to women who has utilised IVF.

An attitude is usually defined as “favourable or unfavourable disposition toward social objects, such as people, places, or policies” [13]. In this investigation, we are interested in attitudes towards individuals who underwent IVF rather than towards the procedure itself, because the attitudes may predict actions towards the attitude object (e.g., stigmatization and discrimination vs acceptance and care). Our focus is specifically on women, because while infertility treatment concerns the couple as a whole, mainly women are identified with infertility treatment in Poland [14–16]. Specifically, according to medical data [17] as well as the public opinion, the assessment of procreation capacities of women is by far crucial for becoming pregnant and significantly easier than the assessment of the procreation capacities of men (ultrasonography in comparison to examination of semen) [17]. Therefore women are associated with greater responsibility for fertility treatment and are more stigmatized, as being a mother is one of the most important social roles in traditional societies [18] . As we intended to prime a stronger connection between the attitude and the attitude object, we decided to analyse attitudes towards women in this preliminary research. Furthermore, predicting behaviour based on attitudes is possible if the attitude object is specific [19, 20].

An attitude is relatively stable and consists of three components: behavioural, emotional, and cognitive. The behavioural aspect relates to a person’s experience (past and present) with the attitude object. Many of the attitudes people hold are products of direct positive or negative experiences with the object. Minimising negative attitude towards a certain object or group is referred to as the contact hypothesis [21]. It is well-established that contact leads to lowering the level of prejudice and improves overall intergroup relationships [22]. Simultaneously, the behavioural component of a positive attitude is expressed by tolerance acceptance of the attitude object. Studies indicate that the degree of personal experience (behaviour) with the attitude object, as well as the importance and accessibility of the attitude object influence attitude development and allow to predict the effect of attitude on behaviour [23]. Previous research has identified several predictors of attitudes towards controversial social issues and/or minority groups (e.g., sexuality or climate change), some of which include knowing a person who is a part of a minority group or has unpopular beliefs [24]. We argue, therefore, that a lack of experience with the attitude’s “object” may result in negative attitudes towards people utilising IVF.

Therefore, the current study predicts that:

### Hypothesis 1

Contact with a person who underwent IVF is a positive predictor of the attitude towards women who utilised IVF.

Emotional reaction to an object or situation is another source of attitudes. Moral judgment depends on emotions and is usually made automatically and intuitively [25]*.* People vary in the extent to which they endorse certain morals. Moral Foundations Theory provides an opportunity to better understand moral diversity in judgments [26]*.* According to the Moral Foundation Theory, there are five moral domains that provide information whether an action is moral or immoral: care/harm (whether the act protects or hurts others), fairness/cheating (whether the action renders justice), loyalty/betrayal (whether the action is congruent with standing with one’s group), authority/subversion (whether the action submits to tradition and legitimate authority), and sanctity/degradation (whether the action is seen as not repugnant). Studies indicate that expressed moral codes allow to predict attitudes towards controversial social issues, including in vitro fertilization [27]. We argue that, by extension, moral foundations explain attitudes towards women using IVF*.* As previous research indicates [27], moral judgement regarding controversial social issues often involve care/harm moral foundation as a positive predictor, and sanctity/degradation moral foundation as a negative predictor. Therefore, the second research hypotheses driving this current study was:

### Hypothesis 2

Moral foundations of care/harm is a positive (a), whereas sanctity/degradation is a negative (b) predictor of the attitude towards women who underwent IVF.

Finally, knowledge or beliefs (cognition) are another source on which an attitude is based. Some attitudes rely on important information that we hold regarding the attitude object. The cognitive information source has been described as influential and more powerful for strong attitudes [28]. Knowledge shapes attitudes towards many matters that may seem controversial, e.g., mental illness [29] or vaccination [30]. Identification of proper knowledge as a substantial source of the attitude may provide an opportunity to utilise this source of information in changing the attitude [31]. The final research hypotheses driving this current research was:

### Hypothesis 3

Knowledge about the IVF procedure is a positive predictor of the attitude towards a woman who underwent IVF.

Overall, the current study seeks to understand the different sources of attitudes towards women who undergo IVF process. In particular: what is the relative role of personal experience - contact (behaviour), moral foundations (emotion), and knowledge (cognition) in forming attitudes towards women who utilise IVF?

## Method

### Participants and procedure

Participants were recruited via the SONA research management system. The participant pool consists of students with diverse background of one university which has campuses across five major towns in Poland. The participant pool consists of undergraduate students that continue education after high school, as well as more professionally experienced master or post-graduate students. Participation was anonymous and voluntary, and participants earned required course credit for participation. The topic of the study was described as attitudes, opinions and knowledge about In-Vitro Fertilization in Poland. Overall, 817 participants agreed to take part in this study (692 women, 118 men, 7 unreported). Based on calculations made using G*Power [32], this final sample provided power of 0.95 to detect a small effect size of f^*2*^ = 0.02 for Hypotheses 1 to 3. The age of the participants ranged between 18 and 60 with mean age 26 (SD = 8). Thirty per cent of participants had higher education (BA or MA). More than half of the sample lived in cities with more than 100,000 citizens (56%). Most participants did not have children (82%). Of those, who had children (*n* = 150), 64% did not experience problems with conception.

#### Measures

To classify the contact variable, we had to identify if the study participants had personal connections with a person who underwent the IVF procedure. Thus, the respondents were simply asked if they personally knew someone who had undergone IVF. Two hundred and fifty-four individuals responded ‘yes’ and five hundred sixty-three participants responded ‘no’. These answers were coded as 1 and 0, respectively.

To measure moral foundations we applied the Polish adaptation [27] of Moral Foundation Questionnaire (MFQ) [33]. This tool allows for measuring five moral foundations postulated by Moral Foundations Theory: care/harm (“Compassion for those who are suffering is the most crucial virtue”; α = .71), fairness/cheating (“Justice is the most important requirement for a society.”; α = .63), loyalty/betrayal (“People should be loyal to their family members, even when they have done something wrong.”; α = .73), authority/subversion (“Respect for authority is something all children need to learn.”; α = .73), and sanctity/degradation (“I would call some acts wrong on the grounds that they are unnatural.”; α = .77). In Part I – Moral Relevance – participants report how relevant are each of the presented grounds for them (e.g., “Whether or not someone suffered emotionally”), using an answering category from 1 to 6, where 1 indicates not at all relevant, whole 6—extremely relevant. In Part II – Moral Judgments – respondents indicate the degree to which they agree with the statements (e.g., “One of the worst things a person could do is hurt a defenceless animal”), using the following response options: strongly disagree, moderately disagree, slightly disagree, slightly agree, moderately agree, strongly agree. Scores for part I and II form composite score for each of the five moral foundations.

To identify the level of knowledge about IVF, a test was constructed. The set of questions was prepared in cooperation with one of the Polish IVF clinics and an association for parents undergoing IVF. The knowledge test consisted of 62 questions concerning all the aspects of IVF procedure such as: general information about infertility and IVF procedure (e.g.,” IVF is painful for the women undergoing the procedure”), pregnancy and its course (e.g.,” From the biological point of view the IVF pregnancy does not differ from a pregnancy obtained without medical intervention”), IVF conceived child development (e.g., “Children conceived through IVF do not suffer from developmental disorders more often than children conceived spontaneously”). There were two types of answers for the questions: true/false and multiple choice. The participant gained 1 point for each correct answer and the summative total derived the knowledge test score. The test was pre-tested in a pilot study (*N* = 190), which showed that the results ranged between 20 and 60. The scores were moderately left-skewed (*M* = 44).

To measure the *attitude towards women who utilise IVF* a modified version of Bogardus Social Distance Scale [34] was used. Each participant was asked to answer a set of six questions concerning a hypothetical woman: “Would you be willing to have a woman undergoing IVF procedure to: 1) live in your city, 2) live in your neighbourhood, 3) be your nearest neighbour, 4) be your colleague, 5) be your friend, 6) marry your son” (Cronbach’s alpha = .93). The answering categories ranged from 1 (definitely not) to 5 (definitely yes). The score was calculated as the mean responses to all questions. Higher score reflected a more positive attitude of the participant towards the hypothetical attitude object: a woman undergoing an IVF.

## Results

### Analysis strategy

Data were analysed using hierarchical regression analysis, wherein predictors are introduced separately in each step to evaluate the contributions of predictors above and beyond previously entered predictors, as a means of statistical control, and for examining incremental validity. A significant F-change and an increase in R-square means that the variables added in that step significantly improved the prediction. The dependent variable was the attitude towards a woman who underwent IVF. In Step 1, we introduced contact with a person who underwent IVF, in Step 2 we added moral foundations (all five codes at once), and in Step 3 we added knowledge about IVF (test score), as predictors.

Next, the outcome was categorized into two tallies (positive vs negative attitude) for a more informative result. Positive attitude was coded as any mean score of 4 or higher and used as an outcome in a step-wise logistic regression. The steps were identical to those of regular regression described above.

### Hypotheses testing

Table 1 displays the means, standard deviations, and intercorrelations of the study variables.

**Table 1:** Means (M), standard deviations (SD), and zero-order correlations between variables

Variable	M	SD	(1)	(2)	(3)	(4)	(5)	(6)	(7)
Attitude towards a woman who underwent IVF (1)	4.75	0.62	–						
Contact ^a^ (2)	–	–	.06	–					
Moral foundations									
*Care/harm (3)*	5.16	0.67	.29***	.02	–				
*Fairness/cheating (4)*	4.77	0.68	.22***	.04	.70***	–			
*Loyalty/betrayal (5)*	3.49	0.89	−.07	.01	.23***	.33***	–		
*Authority/subversion (6)*	3.23	0.92	−.11**	.08*	.17***	.26***	.74***	–	
*Sanctity/degradation (7)*	3.53	1.03	−.12**	.11**	.31***	.35***	.57***	.68***	–
Knowledge about IVF (test score) (8)	38.59	6.43	.32***	.09*	.23***	.14***	−.26***	−.27***	−.19***

*Note. N* = 817

^a^ Coding: 0–no, 1–yes

* *p* < .05, ** *p* < .01, *** *p* < .001

To test our hypotheses, we performed the hierarchical regression analysis. Table 2 presents the results of each model.

**Table 2:** Hierarchical regression with contact (Step 1), moral foundations (Step 2), and knowledge about IVF procedure (Step 3) predicting an attitude towards a woman who underwent IVF

	Model 1		Model 2		Model 3	
Variable	β	t	β	t	β	t
Contact^a^	0.06	1.83	0.08	2.45*	0.06	1.79
Moral foundations						
*Care/harm*			0.26	5.71***	0.20	4.47***
*Fairness/cheating*			0.16	3.33**	0.14	2.95**
*Loyalty/betrayal*			−0.04	−0.70	0.01	0.13
*Authority/subversion*			−0.01	−0.26	0.01	0.21
*Sanctity/degradation*			−0.23	−5.00***	−0.21	−4.48***
Knowledge about IVF (test score)					0.22	6.10***
*R*^*2*^ (adjusted R)	.004 (.003)		.149 (.143)		.186 (.179)	
Δ*R*			.145		.037	

Note. N = 817

^a^ Coding: 0–no, 1–yes

* *p* < .05, ** *p* < .01, *** *p* < .001

In Model 1, with contact is a sole predictor of the attitude towards a woman who underwent IVF, the model was not statistically significant, *F* (1, 815) = 3.36, *p* = .067. Knowing someone who underwent IVF was a weak and insignificant predictor of the attitude (*β* = 0.06, *p* = .067). Due to uneven number of cases in groups (*n* = 254 for ‘yes’ and *n* = 563 for ‘no’), we performed an additional non-parametric test (Mann-Whitney U) that allowed us to reject the null hypothesis; those, who knew a person who underwent an IVF (*M* = 4.81, SD = 0.52) had a more positive attitude towards a hypothetical woman undergoing IVF than people who did not know such person (*M* = 4.72, SD = 0.65), *U* = 64,434.00, *p* = .004. This result supported Hypothesis 1.

Adding moral foundations in step 2 allowed for a significant increase in variance explained by c. 14%. The model predicting the attitude towards a woman who underwent IVF was statistically significant, *F* (6, 810) = 23.66, *p* < .001. As Table 2 demonstrates, supporting Hypothesis 2a, the attitude towards a woman who underwent IVF was predicted positively by care/harm (*β* = .26, *p* < .001). In line with Hypothesis 2b, the moral code of sanctity/degradation (*β* = −.23, *p* < .001) was a negative predictor of the attitude towards a woman who underwent IVF. We also found a positive significant link for the fairness/cheating (*β* = .16, *p* = .001) foundation. The other two moral foundations did not allow to predict the attitude toward the woman who underwent IVF significantly.

In Model 3, we added the scores obtained in the knowledge test about IVF as an additional predictor, which resulted in additional variance explained in the attitude towards a woman who underwent IVF (increase by 3.7%). The model was statistically significant, *F* (7, 809) = 26.49, *p* < .001. As predicted by Hypothesis 3, more knowledge about IVF was linked with a more positive attitude, *β* = .26, *p* < .001. Contact was no longer a significant predictor of the attitude (*β* = .06, *p* = .074). Care/harm (*β* = .20, *p* < .001) and fairness/cheating (*β* = .13, *p* = .003) foundations were positive, and the sanctity/degradation foundation was a negative predictor of the attitude towards a woman who underwent IVF (*β* = −.21, *p* < .001).

In the last step we performed the logistic regression with positive attitude as outcome (see Table 3). The final model (Model 3) shows that knowing someone who underwent IVF (i.e., contact) increased the odds of developing a positive attitude towards a hypothetical woman who underwent IVF (OR = 1.97). Next, strong care/harm (OR = 2.59) and fairness/cheating (OR = 2.22) moral foundations were linked with higher odds of a positive attitude, whereas strong sanctity/degradation moral foundation decreased the odds of a positive attitude (OR = 0.33). Finally, in line with the hypotheses, as test scores (knowledge) increased by 1, the probability of a positive attitude increased by 1.12 times.

**Table 3:** Logistic regression with contact (Step 1), moral foundations (Step 2), and knowledge about IVF procedure (Step 3) predicting a positive attitude towards a woman who underwent IVF

	Model 1			Model 2			Model 3		
Variable	B	W	OR	B	W	OR	B	W	OR
Contact^a^	0.29	1.03	1.33	0.65	4.11*	1.92	0.68	3.92*	1.97
Moral foundations									
*Care/harm*				1.27	20.82***	3.56	0.95	10.27**	2.59
*Fairness/cheating*				0.92	9.13**	2.52	0.80	6.28*	2.22
*Loyalty/betrayal*				−0.33	1.73	0.71	−0.15	0.30	0.87
*Authority/subversion*				−0.01	0.00	0.99	0.13	0.23	1.14
*Sanctity/degradation*				−1.15	26.04***	0.32	−1.12	22.91***	0.33
Knowledge about IVF (test score)							0.11	21.98***	1.12
*Nagelkerke’s R* ^*2*^	.003			.296				.351	

Note. *N* = 817

Outcome: Positive attitude (0–no, 1–yes)

B–unstandardized beta coefficient, W–results of Wald test, OR–odds ratio

^a^ Coding: 0–no, 1–yes

* *p* < .05, ** *p* < .01, *** *p* < .001

## Discussion

The aim of the present study was to examine three possible predictors of attitudes towards women who undergo IVF – contact with a person who has undergone IVF (behaviour), moral foundations of participants (emotion), and the level of knowledge (cognition) about the IVF procedure. The results of the study indicate that knowing someone who underwent IVF procedure (i.e., the contact hypothesis) is a weak predictor of an attitude towards women who underwent IVF. Next, we demonstrated the importance of three moral foundations in predicting the attitude: positive links with care/harm and fairness/cheating, and a negative link with sanctity/degradation. Finally, our results point to the importance of knowledge about the IVF procedure; namely, a higher score in the IVF knowledge test predicted positive attitudes towards women who underwent IVF over and above the enlisted moral codes. Below, we explain the contribution of these findings.

The results have indicated that attitudes towards women who have utilised IVF are mostly based on the emotional (moral foundations) and cognitive attitude components (knowledge about the procedure). Moral Foundations Theory argues that people use distinct codes as basis for their judgments [33]; this theory may serve as an explanation of the intensity of discourse around the IVF. Namely, because representatives of opposite views may be less sensitive to one or more of the moral foundations of the opponents, they may perceive their words or behaviour as less legitimate. Some moral codes may be especially important for the attitudes in less secularized countries, such as Poland. According to a 2012 report, 14% of the general public would not use IVF even though they believe that the method should be accessible for the infertile couples. The respondents who reject the possibility of using IVF as a method of treating infertility base their decision on their moral values and beliefs (79%) [6]. With this study we contribute to the knowledge by demonstrating that there are three main codes that participate in perceiving IVF in terms of morality: care/harm, fairness/cheating, and sanctity/degradation. We suggest that these perspectives are important to acknowledge in the discussion around this topic. To build more common ground in communication, people should be aware of the value others place on the moral codes we enlisted.

Attitude theory indicates that the potential to change the attitude may depend upon the component that the attitude is based on [35, 36]. While moral codes relate to values and are harder to change [25], we showed that more knowledge explains attitudes over and above the moral domain. Thus, the main contribution of our research is that cognition, i.e., more education and knowledge, may help shape more positive attitudes towards IVF regardless of one’s moral codes. This pattern provides an opportunity to redress the knowledge deficit and, hence, potentially change attitudes toward the utilisation of IVF for infertility treatment in Poland. Public education campaigns should be directed toward upgrading the level of knowledge of citizens in relationship to infertility as a step towards forming more positive attitudes towards couples who need to undergo IVF as an assisted reproduction treatment. Educational programs in infertility mainly focus on the infertile couples and incorporate coping strategies training, stress reduction, sex therapy, and receiving preparatory information about medical tests or treatment [37]. Our research demonstrates that greater emphasis should be placed on educating the public. Considering the fact that attitudes of the society and the perceived social support may influence the course of infertility treatment [38, 39], it is of utmost importance to develop educational programs to increase knowledge about this procedure, which may shape positive attitudes towards couples who undergo IVF. Focusing on knowledge gaps or correcting erroneous beliefs could help raise better awareness concerning IVF in Poland. Furthermore, more education may contribute to higher readiness for open discussion about infertility treatment; such openness could, to some extent, alleviate stress experienced by the couple undergoing the treatment. It is important to note that whilst the contact with a person who underwent IVF was a weak predictor of the attitude, direct contact may serve as an access point for “gaining” more knowledge about a women who utilize IVF, because attitudes based on experience are more stable [35].

Apart from the above contributions, some limitations need to be addressed. The study is cross-sectional in its design; therefore, the causality between the variables cannot be inferred. Future studies should adopt a longitudinal or intervention design to see how initial knowledge and then further IVF knowledge education contributes to potential changes in attitudes to women who have utilised IVF. Another limitation is convenience sampling, which resulted in a non-representative sample: individuals were recruited among the student participant pool and consisted mainly of female participants (84%). This pattern limits the generalizability of the results for the following reasons. First, higher education may be linked with more acceptance towards IVF; however, this is not uniformly supported by research findings [40]. Second, research has shown that gender plays a role in the acceptance of assisted reproductive methods as women are more sympathetic than men^1^ [41]. Research suggests that women tend to score higher than men on tender-mindedness, which involves nurturance and empathy [42]. These traits may predispose women to be more compassionate about the stress related to infertility, thus, potentially affecting attitudes. Therefore, future studies should address the issue of gender, with the inclusion of more males and individuals with more diverse educational background to better represent the general population and thus effect the relationships currently observed. In addition, most participants in our sample did not have children or experience problems with conception. Yet, the degree of acceptance of IVF may be different depending on such experiences [43], and this may have affected the pattern of our finding. Overall, we believe that this research should be replicated using a nationally representative sample of participants with different educational and socio-economic backgrounds to examine the meaning of identified predictors among different groups.

Also, the study should be extended by including a man or the couple as the attitude object. As mentioned before, our focus was specifically on women, because while infertility treatment concerns the couple, mainly women are identified with infertility treatment in Poland [14–16]. Nevertheless, men are also deeply affected by infertility treatment procedures and social views on their procreation problems have an impact on their health and well-being [44]. Given the scarcity of research that focuses on men and IVF, in future research it is necessary to study attitudes towards men undergoing the IVF procedure. The role of predictors we proposed in our model may also be different if we measured attitudes towards non-traditional IVF users, like single women or homosexual couples. It is possible that in these cases the role of moral foundations may be stronger. In this study, we hypothesized about the importance of contact, i.e., familiarity to a person who underwent IVF, for attitudes. However, we did not control the degree familiarity, i.e., level of closeness and frequency of contacts. Yet, proximity may increase the effects of contact; thus, we propose to control for it in the future research. Next, the current study administered a newly developed IVF knowledge test; however, it had been validated previously in the pilot study and was developed in cooperation with fertility clinic. Finally, it should be noted that with 17.9% of the variance explained in the current study, other factors (such as familial, religious and societal influences) would be important to include. Specifically, considering most of the Polish society are *Roman Catholics*, it would be important to take into account the level of religiosity in further research [43]. Research shows that the level of religiosity, measured by the frequency of religious practices, has a significant impact on the attitude towards IVF. The higher the declarative religiosity, the lower the support for assisted procreation, also for married couples [45]. Furthermore, respondents who reject the possibility of applying IVF as a method of infertility treatment base their decision on their values and beliefs [43]. In view of the above, a measurement of religiosity and such convictions in further research seems justified.

## Conclusion

Mindful of the limitations, the current study provides insight into the basis of attitudes towards women who have utilised IVF. This current study demonstrated that it is cognition and emotion that predict attitudes towards women who have underwent IVF. Thus, our research demonstrates moral foundations that are important in forming attitudes toward people who underwent IVF; these moral codes could be used to facilitate communication about this topic in Poland. Further, based on our research, we underline the importance of further education as an important step in building acceptance toward couples who chose IVF as their assisted reproduction treatment.

## Data Availability

The data and syntax are available online at https://osf.io/phq25/.

## Abbreviations

IVF: In Vitro Fertilization
OR: Odds Ratio

## Footnotes

1 In light of this limitation, we performed additional analyses to examine the role of gender in our models. Gender served as a control variable and was entered in the first step of the stepwise regression analysis. Across all models, gender was not significantly related to the attitudes (all *p*’s > .200). We also checked whether the pattern of results was similar across both genders by testing the models separately for men vs. women. The directions and strengths of the relationships were similar for all predictors except for care/harm moral code, where the link was stronger for male participants.
